# Sugar Metabolisms Altered By Undissociated Forms of Organic Acids Based on the Emergence of [*GAR* ^+^] Cells in *Saccharomyces cerevisiae*

**DOI:** 10.1002/yea.70031

**Published:** 2026-06-08

**Authors:** Koichi Tanabe, Atsuhiko Nakata, Mizuki Hosotani, Jun Shima

**Affiliations:** ^1^ Faculty of Agriculture Ryukoku University Shiga Japan; ^2^ Research Center for Fermentation and Brewing Ryukoku University Shiga Japan; ^3^ Faculty of Food and Agricultural Sciences Fukushima University Fukushima Japan

**Keywords:** [*GAR* ^+^] emergence, glucose repression, organic acids, *Saccharomyces cerevisiae*

## Abstract

The glucose repression system is a mechanism for effective energy acquisition by glucose assimilation in microorganisms. In yeast, *Saccharomyces cerevisiae*, which is known as a prion‐like protein [*GAR* ^+^], is involved in the bypass of glucose repression. It has been reported that the emergence of [*GAR* ^+^] cells was promoted by lactate and acetate. Herein, we examined the promotion of [*GAR* ^+^] emergence by pyruvate (both the end product of glycolysis and a precursor of the synthesis of organic acids), and we compared the results of pyruvate with those of lactate or acetate. Pyruvate, as well as other organic acids, promoted [*GAR* ^+^] emergence in all *S. cerevisiae* strains tested, but the induction patterns of [*GAR* ^+^] emergence by organic acids differed among the strains. The ability of all three organic acids to induce [*GAR* ^+^] was weakened at pH values greater than the p*K*a value of each organic acid, indicating that the undissociated forms of organic acids may promote [*GAR* ^+^] emergence.

## Summary

- [*GAR* ^+^] yeast cells circumvent glucose repression.
- We tested the major organic acids present in sake brewing mash to determine whether they enhance the frequency of [*GAR* ^+^] cell emergence.
- Acetate, lactate, and pyruvate each promoted the [*GAR* ^+^] emergence frequency dose‐dependently.
- An increase in extracellular pH abolished the induction of [*GAR* ^+^] cells by organic acids, suggesting that the undissociated forms of these acids may promote [*GAR* ^+^] emergence.

## 1 Introduction

In heterotrophic microorganisms, cellular energy is acquired primarily by the catabolism of organic substances such as glucose (Nair and Sarma 2021), and the glucose metabolism of the yeast *Saccharomyces cerevisiae* has been clarified (Kayikci and Nielsen 2015; Shashkova et al. 2015). Under glucose‐rich conditions, *S. cerevisiae* uses glucose alone by repressing the metabolic pathway for the utilization of carbon sources other than glucose. The system, which is known as glucose repression, is controlled by the transcription levels of multiple genes involved in the utilization of carbon sources. Under glucose‐poor conditions, the metabolic pathway for sugars other than glucose is activated. The regulation of carbon‐source utilization may directly contribute to the efficient conversion of sugar to ethanol in *S. cerevisiae* cells.

Prion‐like proteins [*GAR* ^+^] epigenetically allow yeast cells to bypass glucose repression, enabling the utilization of multiple carbon sources even in the presence of glucose, whereas yeast cells under glucose repression are denoted as [*gar* ^−^] (Ball et al. 1976; Jarosz et al. 2014; Watanabe and Takagi 2019). This process suggests that a shift to [*GAR* ^+^] from [*gar* ^−^] may allow yeasts to become metabolic generalists and enable them to use various carbon sources (Jarosz et al. 2014). Spontaneous [*GAR* ^+^] emergence has been investigated under normal glucose‐rich growth conditions, and it was demonstrated that the frequency of [*GAR* ^+^] emergence was quite low, ranging from 10^−4^ to 10^−5^ cells/total cells (Brown and Lindquist 2009). Our research group recently demonstrated that the frequency of the spontaneous emergence of [*GAR* ^+^] cells of the *S. cerevisiae* U44 strain, which was isolated from sake (Japanese rice wine) brewed by the kimoto traditional brewing method, was much higher (^~^10^−1^ cells/total cells) than the frequencies observed for industrial and laboratory *S. cerevisiae* strains (Tanabe et al. 2023).

On the other hand, the frequency of [*GAR* ^+^] emergence was greatly increased by coculturing with bacteria, including lactic acid bacteria (LAB) (Jarosz et al. 2014; Jarosz et al. 2014; Ramakrishnan et al. 2016). In addition, Garcia et al. (2016) showed that lactate (p*K*a 3.79) strongly promoted the emergence of [*GAR* ^+^] cells in laboratory strains of *S. cerevisiae*. Acetate (p*K*a 4.74) is also reported to promote [*GAR* ^+^] cell emergence (Ramakrishnan et al. 2016). Lactate is the most abundant organic acid in sake mash during brewing, as lactate is commonly supplied at the beginning of the sake brewing process to prevent the growth of harmful microbes, whereas acetate is thought to be produced by the oxidation of ethanol. Pyruvate (p*K*a 2.50), the third most abundant organic acid in sake mash, was also accumulated in the mash of sake brewing (Takahashi and Kohno 2016). Pyruvate is also known as a key metabolite for the biosynthesis of lactate and acetate in various *S. cerevisiae* strains, including laboratory and industrial strains. These observations suggest that these organic acids present in sake mash are potentially involved in the modulation of the [*GAR* ^+^] emergence ratio of yeast cells.

In this study, we investigated the abilities of lactate, acetate, and pyruvate to promote the [*GAR* ^+^] emergence in laboratory and the sake‐brewing strains of *S. cerevisiae*. We also discuss the correlation of environmental pH and the induction of [*GAR* ^+^] emergence by organic acids.

## 2 Materials and Methods

### 2.1 *S. cerevisiae* Strains and Cultivation Media

The *S. cerevisiae* strains used in this study are listed in Table 1. We used yeast extract peptone dextrose (YPD) medium for the cultivation of *S. cerevisiae* unless otherwise stated; this medium is comprised of 2% glucose, 1% yeast extract, and 2% peptone (the latter two from Becton Dickinson, Sparks, MD, USA). Glycerol glucosamine medium (GGM) (1% yeast extract, 2% peptone, 2% glycerol, and 0.05% glucosamine) was used to select [*GAR* ^+^] cells. Yeast extract peptone glycerol (YPG) medium (1% yeast extract, 2% peptone, and 2% glycerol) was used to count the viable cells in each sample.

**Table 1:** The *S. cerevisiae* strains used in this study.

Strain	Description	Source or reference
S2888C	Laboratory strain	(Goffeau et al. (1996))
X2180	Laboratory strain	(Roncero et al. (1988))
701	Commercial strain for sake brewing	National Research Institute of Brewing, Japan (Akao et al. 2011; Tomimoto et al. 2017)
901	Commercial strain for sake brewing	National Research Institute of Brewing, Japan
U44	Isolate from the kimoto brewing of sake	(Tanabe et al. (2023))

### 2.2 Assay for the Spontaneous Emergence of [*GAR* ^+^]

The spontaneous emergence of [*GAR* ^+^] cells was assayed using the method described by Brown and Lindquist (Brown and Lindquist 2009). A preculture of each *S. cerevisiae* strain was prepared using the liquid YPD medium with shaking (180 rpm) at 30°C for 14–18 h. The preculture was diluted with 5 mL of fresh YPD medium to OD_600_ = 0.1 and cultivated with shaking (180 rpm) at 30°C for 6 h. Logarithmic cells (1 mL) in the culture were washed twice with phosphate‐buffered saline (PBS) and spread onto GGM agar after proper dilution. After 5 days of incubation at 30°C, the colonies were counted as spontaneously emerged [*GAR* ^+^] cells. The data are expressed as those after normalization using the total viable cell count on the YPG medium.

### 2.3 Assay of [*GAR* ^+^] Emergence Induction By Acetate, Lactate, and Pyruvate

The conditions for the cultivation and preparation of logarithmic cells were the same as those used in the assay for the spontaneous emergence of [*GAR* ^+^]. After the cells were prepared, they were spread onto GGM agar containing 10 mM acetate, lactate, or pyruvate. After 48 h incubation at 30°C, the colonies were counted as organic acid‐promoted [*GAR* ^+^] cells. The data presented are those after normalization using the total viable cell count on the YPG medium.

### 2.4 Effects of pH Changes on [*GAR* ^+^] Emergence

GGM agar, including acetate, lactate, or pyruvate at 10 mM and various pH values (pH 4.0–8.0), was prepared using HCl or NaOH. The emergence of [*GAR* ^+^] cells on the agar media was assessed.

#### 2.4.1 Assay to Determine the Correlation Between [*GAR* ^+^] Emergence and the p*K*a Values of Organic Acids

The average [*GAR* ^+^] emergence rate in the presence of 1.25 mM organic acid (acetate, lactate, or pyruvate) from three independent experiments was plotted with regard to the acids' corresponding p*K*a values.

### 2.5 Spot Assay

The growth enhancement of yeast cells by organic acids was evaluated based on a previous report (Tanaka et al. 2012). Briefly, yeast cells grown in YPD medium to the stationary phase were serially diluted with PBS and 5 μL of serial ten‐fold dilutions of cell suspensions at concentration of 1, 10^−1^, 10^−2^, 10^−3^, and 10^−4^ were spotted onto YPG or YPG containing 5 mM organic acid (acetate, lactate, or pyruvate) agar medium and incubated at 30°C for 24 h.

The induction of [*GAR* ^+^] cells by lactate was also confirmed by a spot assay. Yeast colonies grown on GGM containing 5 mM lactate were directly suspended in PBS, and the cell density of each suspension was determined by a microplate reader (Infinite M1000 Pro, Tecan Japan Co., Kanagawa, Japan). The cell suspension was serially diluted and spotted on GGM agar medium. The cells grown on YPD agar plates were also tested as control [*gar* ^*−*^] cells.

### 2.6 Statistical Analysis

The results are expressed as the mean ± standard deviation from three independent experiments. Statistical analyses were performed using one‐way analysis of variance (ANOVA), followed by the Tukey–Kramer test for multiple comparisons among groups. A *p*‐value < 0.05 was considered significant.

## 3 Results

### 3.1 Spontaneous Emergence of [*GAR* ^+^] Cells of the *S. cerevisiae* Strain

**Figure 1:**
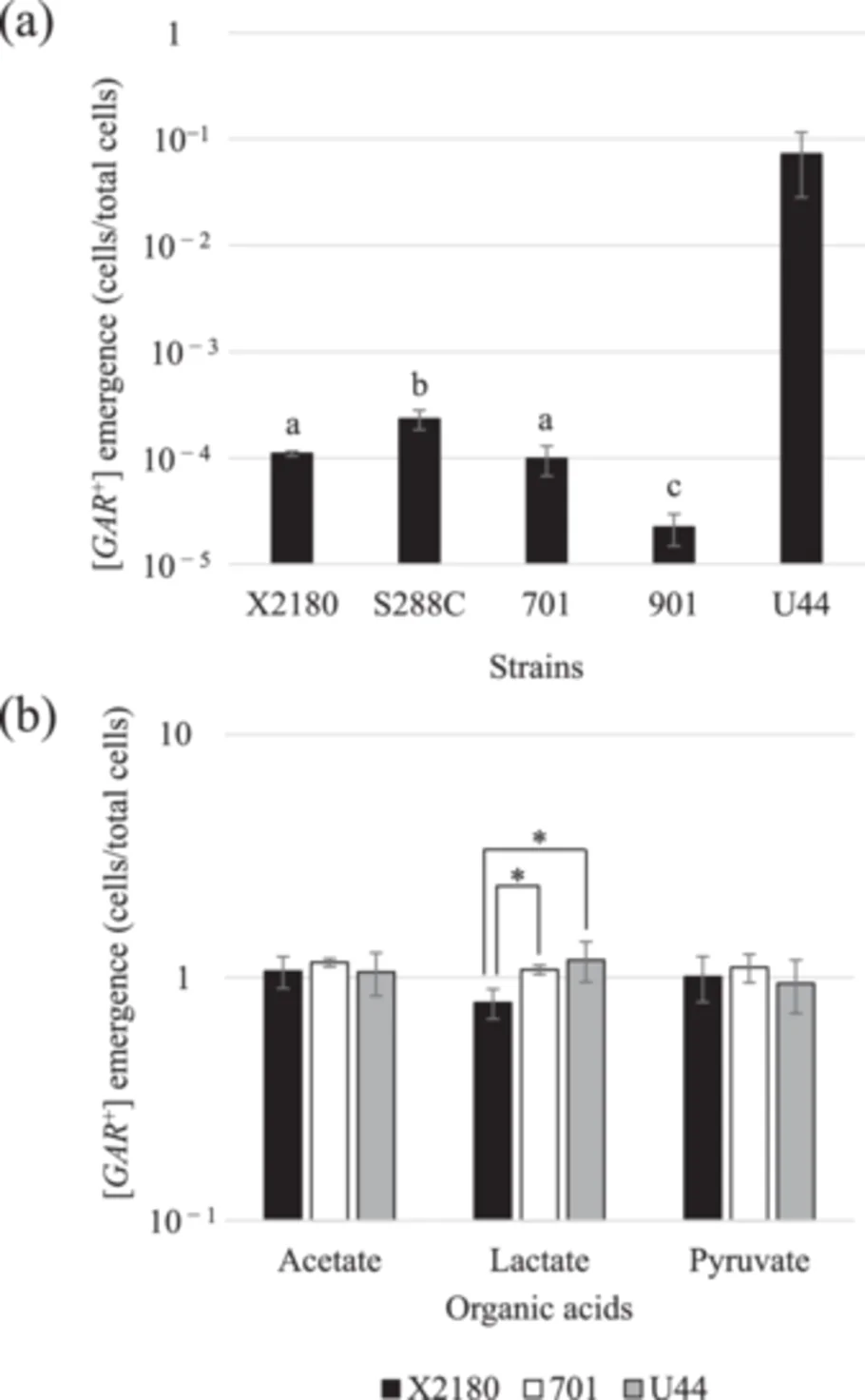
Organic acids promote the emergence of [*GAR* ^+^] cells in *Saccharomyces cerevisiae* strains. (a) The spontaneous [*GAR* ^+^] cell emergence rate; no supplement. Yeast cells in the logarithmic growth phase were spread onto GGM agar, and the plates were incubated for 5 days at 30°C. Colonies appearing on the GGM were counted as [*GAR* ^+^] cells and normalized by the number of colonies appearing on the YPG agar medium. Bars: average data from three biological replicates. Error bars: SD. Bars not sharing a common letter are significantly different at *p* < 0.05. (b) The [*GAR* ^+^] cell emergence rate in the presence of the indicated organic acid (acetate, lactate, or pyruvate) at 10 mM. Yeast cells were spread onto GGM agar supplemented with organic acids, and the plates were incubated for 48 h at 30°C. Colonies appearing on the medium were counted as [*GAR* ^+^] cells. Black bars: strain X2180. White bars: strain 701. Gray bars: strain U44. Each bar is the average of data from three biological replicates. Error bars: SD. *p < 0.05.

Earlier studies described the spontaneous emergence of [*GAR* ^+^] cells that depended on the genetic background of the *S. cerevisiae* strains used (Jarosz et al. 2014; Tanabe et al. 2023; Watanabe et al. 2018). In the present study, we examined the spontaneous [*GAR* ^+^] emergence ratio of several *S. cerevisiae* strains, as depicted in Figure 1a. The laboratory strains and the sake brewing strains (other than strain U44) showed a spontaneous [*GAR* ^+^] emergence ratio of 10^−5^ to 10^−4^ cells/total cells. Strain U44 showed higher spontaneous [*GAR* ^+^] emergencies (approx. 10^−1^ ells/total cells) as reported (Tanabe et al. 2023). We used *S. cerevisiae* strains X2180, 701, and U44 for further analysis.

### 3.2 Induction of [*GAR* ^+^] Cells by Various Organic Acids

We compared the ability of different organic acids at 10 mM to promote the emergence of [*GAR* ^+^] cells (Figure 1b). The levels of [*GAR* ^+^] emergence promoted by acetate, lactate, and pyruvate were nearly 1 cell/total cells in all tested strains, which suggests that 10 mM organic acids tested in this study have the ability to fully promote the [*GAR* ^+^] emergence ratio in the tested yeast strains. Colonies appeared on GGM agar medium supplemented with organic acids after 48 h of incubation, whereas yeast cells required approximately 5 days to form colonies on GGM agar medium alone. The pH value of the media, including each organic acid, was approximately 5.3.

### 3.3 Dose Dependence of Organic Acids in the Promotion of [*GAR* ^+^] Emergence

**Figure 2:**
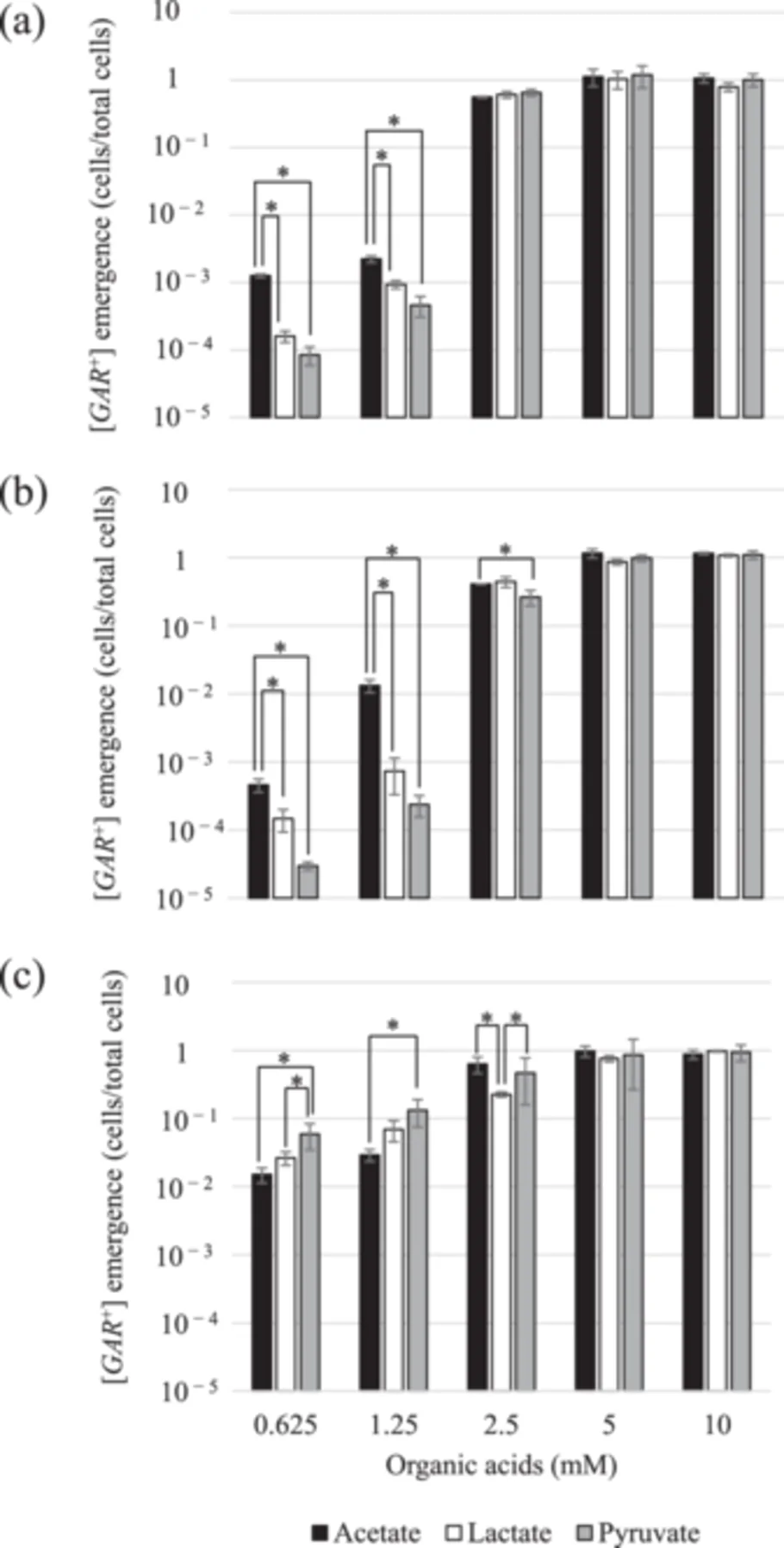
Dose‐dependent promotion of the emergence of [*GAR* ^+^] cells by organic acids. Yeast cells were spread onto GGM agar including the indicated concentration of organic acids, and the plates were incubated for 48 h at 30°C. Colonies appearing on the plates were counted as [*GAR* ^+^] cells and normalized by the number of colonies appearing on YPG agar medium. The [*GAR* ^+^] cell emergence rates of strains (a) X2180, (b) 701, and (c) U44 in the presence of the indicated concentrations of organic acids were determined. *Black bars:* acetate. *White bars:* lactate. *Gray bars:* pyruvate. Each bar is the average of data from three biological replicates. Error bars: SD. *p < 0.05.

We investigated the dose dependence of organic acids in the promotion of [*GAR* ^+^] emergence ratio in strain X2180 (Figure 2a), strain 701 (Figure 2b), and strain U44 (Figure 2c). Strains X2180 and 701 showed a similar concentration‐dependent increase in [*GAR* ^+^] cell emergence. Under the conditions using organic acids at < 2.5 mM, the emergence ratio of [*GAR* ^+^] cells was greatly decreased. The increase in [*GAR* ^+^] cell emergence by acetate was significantly higher than that of pyruvate and lactate in strains X2180 and 701 (Figure 2a,b). In contrast, the [*GAR* ^+^] promotion ability of each organic acid was relatively maintained at a high level in U44.

**Figure 3:**
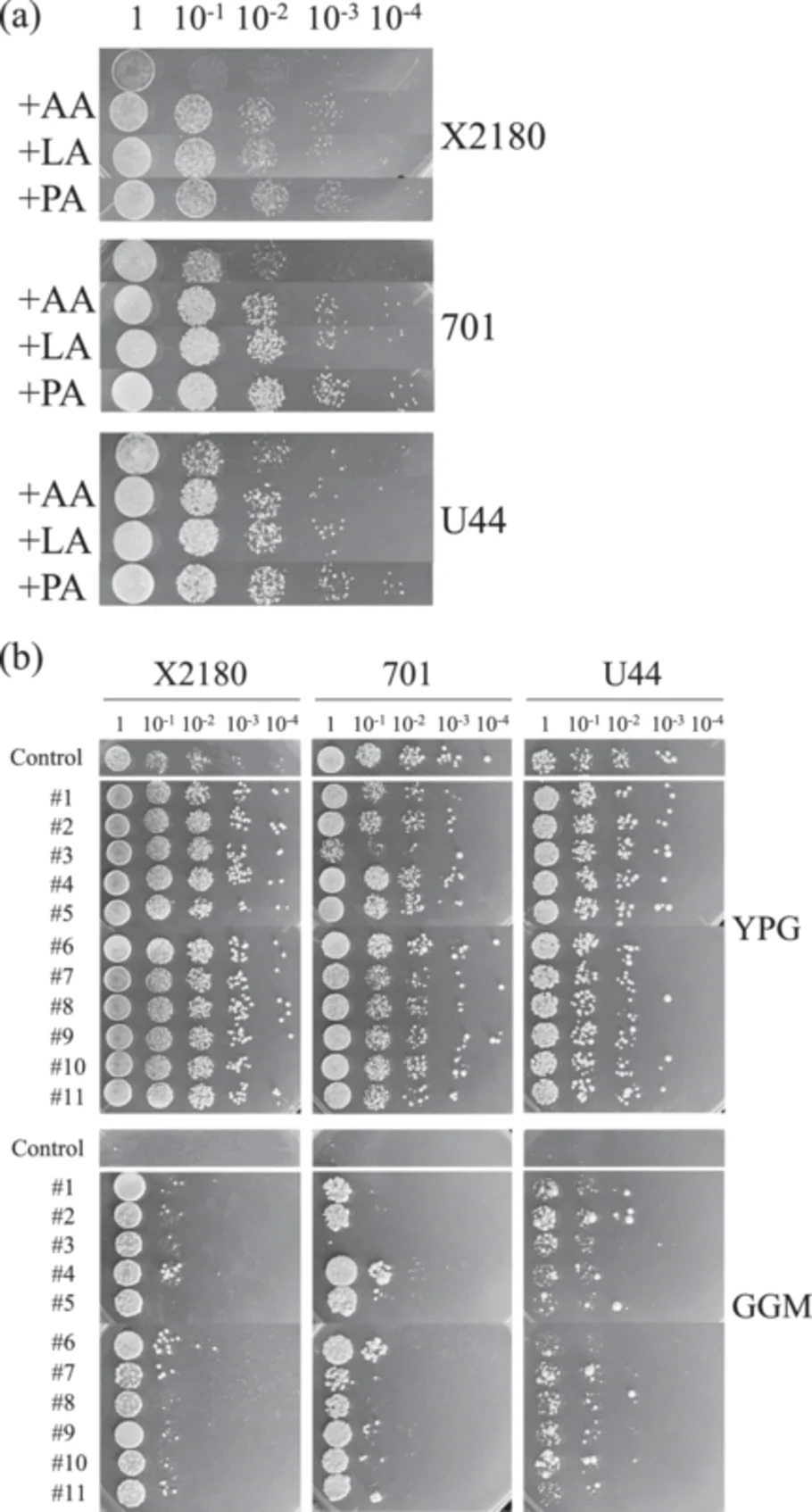
Organic acids promote both yeast growth and the emergence of [*GAR* ^+^] cells. (a) Serially diluted suspensions of yeast cells were spotted on YPG agar medium containing the indicated organic acid (acetate; +AA, lactate; +LA, and pyruvate; +PA) at 5 mM. The agar medium was incubated for 24 h at 30°C. The dilution factors are indicated at the top of the panel. (b) Eleven colonies of each strain that appeared on GGM agar containing 5 mM lactate were randomly picked up and serially diluted. The colonies of each strain that appeared on YPD medium were used as control [*gar* ^*−*^] cells. Serially diluted suspensions of yeast cells were spotted on YPG agar or GGM agar medium and incubated at 30°C for 1 day or 5–7 days, respectively. The dilution factors are indicated at the top of each panel.

It was possible that the addition of organic acids supported the growth of the yeast cells on GGM agar medium and resulted in an increase in the number of colonies on the agar plates. To exclude this possibility, we performed a growth assay of the yeast strains on YPG agar medium containing each organic acid at 5 mM. The addition of organic acids slightly enhanced the growth of strains 701 and U44, whereas more definite growth enhancement was observed in strain X2180 by organic acids (Figure 3a).

We next investigated whether the clones isolated from GGM medium containing an organic acid retained the [*GAR* ^+^] phenotype. Eleven clones of each strain were randomly selected from colonies grown on GGM medium containing 5 mM lactate and then spotted on GGM agar medium (Figure 3b). All of the clones tested in this assay showed significant growth on GGM agar, whereas no growth was observed with the control [*gar* ^*−*^] cells (Figure 3b). These results suggest that lactate induced the conversion of yeast cells from [*gar* ^*−*^] to [*GAR* ^+^] on GGM agar medium.

#### 3.3.1 Effects of Environmental pH on the Emergence of [*GAR* ^+^] Promoted By Organic Acids

**Figure 4:**
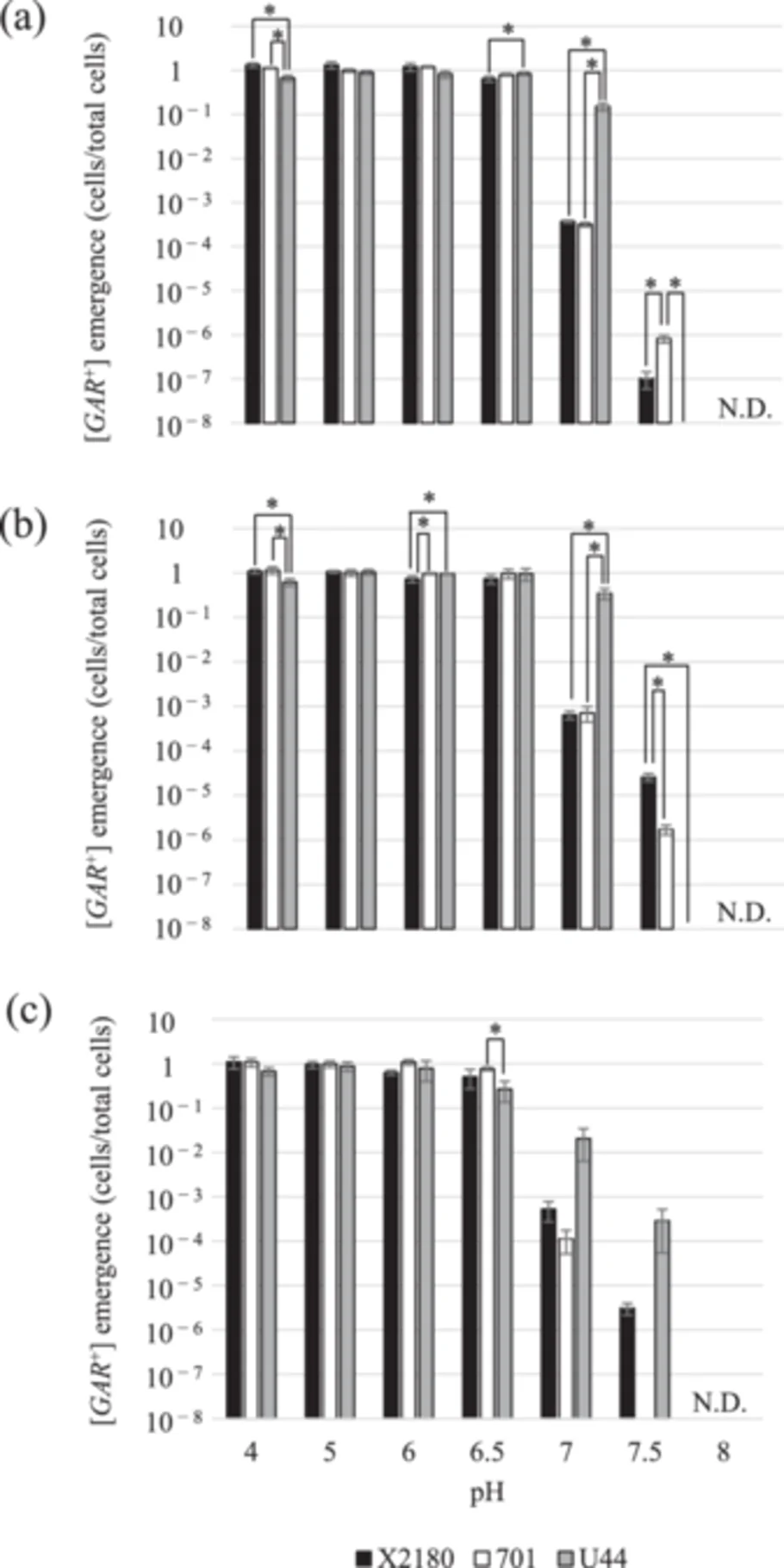
High pH abolished the promotion of the [*GAR* ^+^] cell emergence rate by the organic acids: (a) acetate, (b) lactate, and (c) pyruvate. Yeast cells were spread onto GGM agar containing organic acid at 10 mM under the indicated pH conditions, and the plates were incubated for 48 h at 30°C. Colonies appearing on the GGM plates were counted as [*GAR* ^+^] cells and normalized by the number of colonies appearing on YPG agar medium. *Black bars:* strain X2180. *White bars:* strain 701. *Gray bars:* strain U44. Each bar is the average of data from three biological replicates. Error bars: SD. *p < 0.05.

We investigated the effects of environmental pH values on the [*GAR* ^+^] promotion by acetate (Figure 4a), lactate (Figure 4b), and pyruvate (Figure 4c) at 10 mM. High levels of promotion of [*GAR* ^+^] emergence ratio were observed at pH values < 7, whereas at pH values > 6.5, the promoted [*GAR* ^+^] emergence ratios were markedly decreased, indicating that the dissociation state of the organic acids is crucial for the promotion of [*GAR* ^+^] emergence ratio by organic acids. We further analyzed [*GAR* ^+^] promotion and the p*K*a values of organic acids.

### 3.4 Correlation Between [*GAR* ^*+*^] Promotion Ability and the p*K*a of Organic Acids

**Figure 5:**
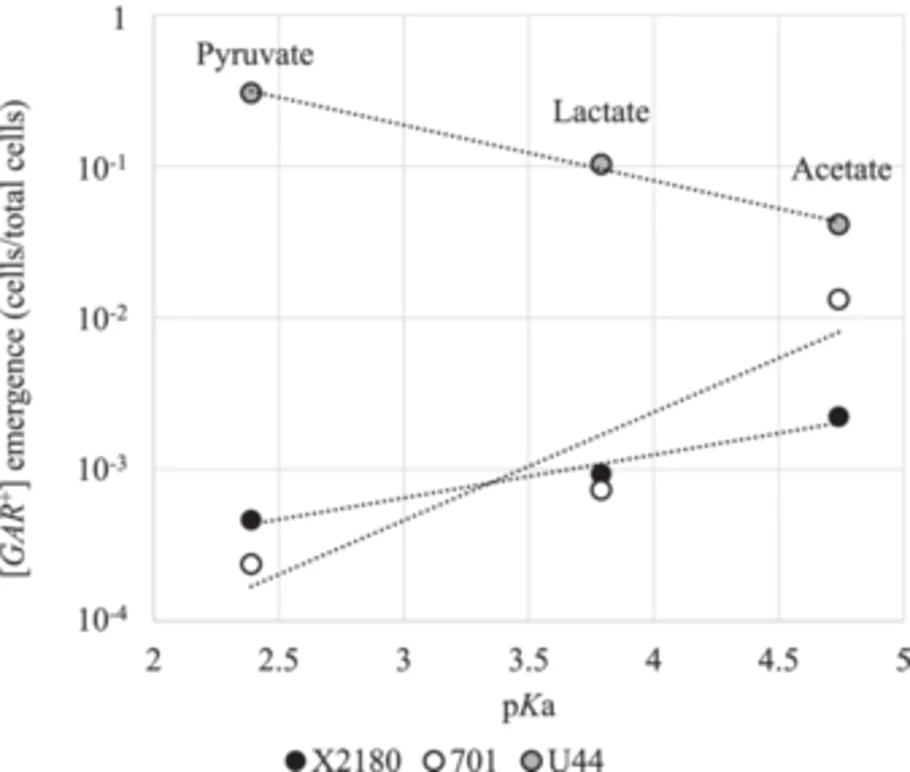
The correlation between the p*K*a value of the tested organic acid and the promotion of [*GAR* ^+^] cell emergence. The average [*GAR* ^+^] cell emergence rate in the presence of each organic acid at 1.25 mM from three independent experiments was plotted with respect to the corresponding p*K*a values.

Organic acids are converted into their dissociated forms at pH values above their p*K*a. We prepared plots of the data of [*GAR* ^+^] emergence ratios in the presence of 1.25 mM of each organic acid versus p*K*a values in *S. cerevisiae* strains X2180, 701, and U44 (Figure 5). Strains X2180 and 701 showed a similar pattern of correlation between [*GAR* ^+^] promotion ability and the p*K*a of organic acids. The p*K*a values and the [*GAR* ^+^] emergence values showed a positive correlation (*R* ^2^ = 0.9746 for strain X2180, *R* ^2^ = 0.9779 for strain 701), suggesting that a higher ratio of undissociated organic acid may be more effective in promoting [*GAR* ^+^] emergence. In contrast, a negative correlation (*R* ^2^ = 0.9983) was observed between p*K*a and [*GAR* ^+^] promotion ability in strain U44, indicating that the mechanism of [*GAR* ^+^] emergence in this strain may be different from those of other *S. cerevisiae* strains.

## 4 Discussion

Lactate and acetate were thought to be cross‐kingdom communication tools between *S. cerevisiae* and bacteria, including LAB and acetic acid bacteria, based on [*GAR* ^+^] promotion (Garcia et al. 2016; Ramakrishnan et al. 2016). In the present study, we carefully compared the frequency of [*GAR* ^+^] emergence promoted by lactate, acetate, and pyruvate with the use of five *S. cerevisiae* strains (because of the potential differences in responses to organic acids among the strains). The data illustrated in Figure 1b suggests that the [*GAR* ^+^] promotion abilities of lactate and acetate at a high concentration (10 mM) were at comparable levels in all of the strains under non‐pH‐adjusted conditions (around pH 5.3), which indicates that the response mechanisms to organic acids are conserved among *S. cerevisiae* strains.

We also investigated pyruvate, a precursor for lactate and acetate biosynthesis, as it may have additional roles in [*GAR* ^+^] promotion. Pyruvate is also of interest as it is one of the most abundant organic acids in sake mash (Takahashi and Kohno 2016). The data shown in Figure 1b suggest that 10 mM pyruvate can fully promote [*GAR* ^+^] emergence. Various weak organic acids can induce promotion activities by changing the intracellular pH of *S. cerevisiae* (Ullah et al. 2012) (Thomas et al. 2002). Our search of relevant literature suggests that the present study is the first to investigate whether pyruvate promotes [*GAR* ^+^] emergence. However, an organic acid itself can be utilized as a carbon source for the growth of yeast cells (White and Munns 1950). We observed that acetate, lactate, and pyruvate enhanced the growth of the tested strains on YPG agar medium (Figure 3a). Organic acids may also have partly accelerated yeast cell growth on GGM medium, as colonies were observed within 48 h on GGM medium supplemented with organic acids, whereas approximately 5 days were required for visible colony formation on GGM agar medium alone. Although the extent of growth varied among the clones that we examined, the isolated cells grown on GGM containing 2.5 mM lactate generally exhibited better growth on GGM agar than control [*gar* ^−^] cells (Figure 3b). With X2180 and U44, the isolated clones obtained from GGM medium containing 5 mM pyruvate also grew well on GGM agar (data not shown). These results clearly suggest that the significant increase in colony numbers on GGM containing organic acids reflected the conversion of yeast cells from [*gar* ^*−*^] to [*GAR* ^+^].

It should be noted that clones isolated from GGM containing either lactate grew better than control [*gar* ^−^] cells on GGM plates without organic acids, however their growth on GGM was still weaker than that on YPG plates (Figure 3b). This result suggests that not all, but a fraction of cells exhibited [*GAR* ^+^] phenotype. It also indicates the possibility that the [*GAR* ^+^] phenotype was unstable during growth on GGM agar plates in the absence of organic acids. It should also be noted that the number of colonies on GGM agar plates did not increase when yeast cells (X2180, 701, and U44) were pre‐incubated in YPD liquid medium containing 10 mM lactate (data not shown). Furthermore, no previous studies have demonstrated that the addition of organic acids to liquid medium induces [*GAR* ^+^] cells (Garcia et al. 2016; Ramakrishnan et al. 2016). Based on these results and previous reports, it is reasonable to consider that continuous exposure to organic acids is required for maintenance of the [*GAR* ^+^] phenotype. The induction of [*GAR* ^+^] cells by organic acids has only been examined on GGM medium supplemented with organic acids so far. This effect should therefore be investigated under a variety of culture conditions.

We assessed the dose dependence of organic acids on the promotion of [*GAR* ^+^] emergence, and the results suggest that acetate has a greater ability to induce [*GAR* ^+^] emergence in strains X2180 and 701 than lactate or pyruvate at low concentrations (0.625 or 1.25 mM) (Figures 2a and 2b). However, in strain U44, pyruvate showed relatively higher promotion activity than acetate or lactate (Figure 2c). The promotion pattern of [*GA*R^+^] emergence ratio by organic acids differed among the yeast strains, and the p*K*a values of organic acids seemed to affect these differences in promotion. These observations indicate that the chemical forms, dissociated or undissociated, of organic acids are critical for the promotion of [*GAR* ^+^] emergence ratio.

The pH dependence of organic acids in the induction of [*GAR* ^*+*^] emergence was also assessed, and the results showed that the environmental pH affects the promotion of [*GAR* ^*+*^] emergence by any of the three organic acids investigated herein (Figure 3). These results helped us analyze the correlation between the p*K*a values of organic acids and the promotion of [*GAR* ^*+*^] emergence (Figure 4). The positive correlation between p*K*a and the promotion of [*GAR* ^*+*^] emergence in strains X2180 and 701, and the results suggest that the ratio of the undissociated form of organic acids is critical for promoting [*GAR* ^+^] emergence. Undissociated organic acids, rather than dissociated forms, are passively transported into *S. cerevisiae* cells, and these incorporated organic acids may play a role in promoting [*GAR* ^*+*^] emergence. It thus seems likely that the promotion of [*GAR* ^*+*^] emergence occurred due to intracellular acidification. Previous reports have shown that the [*GAR* ^+^] phenotype is transmitted through prion‐based cytoplasmic inheritance and that the plasma membrane H^+^‐ATPase Pma1 is associated with the appearance of [*GAR* ^+^] (Brown and Lindquist 2009). Undissociated organic acids are taken up by yeast cells, dissociate inside the cells, and decrease the intracellular pH. This decrease in intracellular pH may directly affect the function and/or conformation of Pma1, because Pma1 pumps protons out of the cell to maintain intracellular pH. It is therefore necessary to investigate how organic acids affect the interactions between Pma1 and proteins involved in [*GAR* ^+^] prion formation.

In the *S. cerevisiae* strain U44, we observed a negative correlation between p*K*a and the promoted [*GAR* ^*+*^] emergence by organic acids. As Tanabe et al. (2023) reported, strain U44 was isolated from the kimoto process of sake brewing. The reason(s) for the contrary behavior of this strain remain unclear. Strain U44 might have mechanisms of sensing intracellular organic acids or pH that differ from those of other strains. We expect that whole genome analyses using strain U44 could be useful to further clarify the mechanisms underlying the promotion of [*GAR* ^*+*^] emergence.

Our experiments examined the promotion abilities of [*GAR* ^*+*^] emergence by organic acids in *S. cerevisiae* strains that included laboratory strains and sake‐brewing strains under various conditions. We hypothesized that the mechanisms underlying the promotion of [*GAR* ^*+*^] emergence by organic acids are basically conserved among the strains. Many of the details of [*GAR* ^*+*^] emergence by organic acids remain to be elucidated. It is thus necessary to determine whether organic acids other than acetate, lactate, and pyruvate can promote [*GAR* ^*+*^] emergence. As major determinants of sourness in sake, organic acids are thought to prevent the growth of harmful bacteria during fermentation. Our present findings suggest that these acids also influence ethanol production by promoting the proportion of [*GAR* ^+^] yeast cells in sake mash. The role of such organic acids in improving brewing efficiency should be considered from the perspective of commercial applications (Saerens et al. 2010).

## Author Contributions

Conceptualization: Koichi Tanabe and Jun Shima. Methodology: Koichi Tanabe and Jun Shima. Investigation: Koichi Tanabe, Mizuki Hosotani, and Atsuhiko Nakata. Resources: Koichi Tanabe and Jun Shima. Writing – original draft preparation: Jun Shima. Writing – review and editing: Koichi Tanabe and Jun Shima. Funding acquisition: Koichi Tanabe and Jun Shima. All authors have read and agreed to the published version of the manuscript.

## Ethics Statement

The authors have nothing to report.

## Conflicts of Interest

The authors declare that they do not have any conflicts of interest related to this study.

## Data Availability

The data that support the findings of this study are available from the corresponding author upon reasonable request.
